# Resuming a Dynamic Task Following Increasingly Long Interruptions: The Role of Working Memory and Reconstruction

**DOI:** 10.3389/fpsyg.2021.659451

**Published:** 2021-06-17

**Authors:** Katherine Labonté, François Vachon

**Affiliations:** PACE Laboratory, School of Psychology, Université Laval, Quebec City, QC, Canada

**Keywords:** task interruption, task resumption, dynamic situation, working memory capacity, visual search, multiple object tracking, individual differences

## Abstract

Studies examining individual differences in interruption recovery have shown that higher working memory capacity (WMC) attenuated the negative impact of interruption length on resumption, at least in static contexts. In continuously evolving (or dynamic) situations, however, working memory may not be as central to the effective resumption of a task, especially in the case of long interruptions. One of the main theories of task interruption suggests that dynamic task resumption could depend on a reconstruction of the primary task context, that is, a visual examination of the post-interruption environment. To better define the role of working memory and reconstruction processes in interruption recovery, the current study examined the association between (1) dynamic task resumption following interruptions of various lengths and (2) two cognitive abilities chosen to operationalize the processes under study, namely, WMC and visual search capacity. Participants performed a multiple object tracking task which could be uninterrupted or interrupted for 5, 15, or 30 s while the hidden stimuli continued their trajectory. They also completed tasks measuring the two cognitive abilities of interest. The results revealed that WMC contributed to post-interruption accuracy regardless of interruption duration. On the contrary, visual search capacity was related to faster resumption in the 15-s and 30-s interruption conditions only. Those results show that working memory plays a preponderant role in resumption not only in static, but also in dynamic contexts. However, our study suggests that this mechanism must share the limelight with reconstruction following lengthy interruptions in dynamic settings.

## Introduction

The ability to deal with unexpected task interruptions varies from one person to another, whether in terms of the tendency to resist engaging in an irrelevant interrupting task (Szumowska and Kossowska, 2017), or the capacity to resume a primary task following an interrupting event (Drews and Musters, 2015). Previous studies interested in the association between individual differences and task resumption have centered on static interrupted tasks, which remain unchanged while one’s attention is diverted to a secondary interrupting task (e.g., Drews and Musters, 2015; Foroughi et al., 2016b,c). Reading or writing a text are typical examples of static tasks. These two activities share the common characteristic that their state remains stable during the occurrence of an interruption. In dynamic contexts, however, the situation continues to evolve during an interruption (e.g., St. John and Smallman, 2008; Tremblay et al., 2012; Hodgetts et al., 2014, 2015). Dynamic situations represent activities in which the monitored situation changes without external influence, such as driving or air traffic control. The cognitive processes involved in the resumption of dynamic tasks likely differ from those involved in static task resumption, especially in the case of long interruptions, where pre- and post-interruption scenes can differ greatly (see Salvucci and Taatgen, 2011). However, no study has yet focused on the individual cognitive characteristics associated with successful interruption recovery in dynamic settings. In an attempt to fill this gap in the literature on dynamic task resumption, the current study employed an individual differences approach to uncover the processes involved in the resumption of a dynamic task following interruptions of different lengths.

### Working Memory Capacity and Interruption Recovery

Task interruptions are often detrimental to the execution of an ongoing activity, for instance by lengthening decision time and increasing the likelihood of errors (Bailey and Konstan, 2006; Altmann et al., 2014). So far, most research about the resumption process of a suspended task has focused on factors pertaining to the interrupting task, such as its length (e.g., Monk et al., 2008), its complexity (e.g., Hodgetts and Jones, 2006), and its timing (e.g., Bailey and Iqbal, 2008). A few researchers have also examined how the consequences of interruptions can be modulated by factors specific to the individuals who must deal with the suspension of their ongoing task (e.g., Drews and Musters, 2015; Foroughi et al., 2016c). These studies about individual differences in interruption recovery have identified working memory capacity (WMC) as a major contributor to the effective resumption of a task. More precisely, low-WMC individuals appear to be more vulnerable to errors and increases in response time following an interrupting activity than their high-WMC counterparts (Drews and Musters, 2015; Foroughi et al., 2016a,b,c).

Although there exist a plethora of definitions of working memory (see Cowan, 2017), the attention-control view (c.f. Engle, 2002; Kane and Engle, 2002, 2003) is the most embraced in the literature on task interruptions (e.g., Drews and Musters, 2015; Foroughi et al., 2016c). In his work attempting to clarify the numerous definitions of working memory, Cowan (2017) defines attention-control working memory as:

The use of attention to preserve information about goals and sub-goals for ongoing processing and to inhibit distractions from those goals; it operates in conjunction with short-term storage mechanisms that hold task-relevant information in a manner that does not require attention (p. 1159).

Therefore, high-WMC individuals’ resumption skills would be attributable to their greater capacity to manage interference from the interrupting task while keeping primary task information active in memory during an interruption (Foroughi et al., 2016c). In this regard, previous studies have shown that interrupting tasks are less damageable when they differ from the main (interrupted) task in terms of their content or of the resources needed for their execution (Gillie and Broadbent, 1989; Edwards and Gronlund, 1998; Oulasvirta and Saariluoma, 2004; Ratwani and Trafton, 2008). Such results likely reflect that the interrupting task interferes more with the maintenance and retrieval of information relative to the primary task when these tasks are similar (Li et al., 2012).

The crucial role that both short-term retention of primary task information and interference from interrupting events occupy in the resumption process of a suspended activity has been demonstrated by several authors (e.g., Trafton et al., 2003; Ratwani and Trafton, 2008; Morgan et al., 2013; Gartenberg et al., 2014; Foroughi et al., 2016c; Wilson et al., 2018). The most prevalent explanation for this phenomenon in the literature comes from the memory for goals model (MFG; Altmann and Trafton, 2002), which suggests that behavior is directed by the most active goal (i.e., intention to perform an action or a task) in memory at a given time. When performing a task, the cognitive system strengthens any new relevant goal by frequently sampling it from memory. The activation level of this goal can then quickly reach its peak and surpass that of other goals. Subsequently, gradual decay of the target goal begins in order to make room for other actions necessary to complete the task at hand. This decline of the newly irrelevant goal does not occur instantaneously. Even when a new goal is targeted, it remains possible that an individual erroneously samples one of their previous goals (i.e., performs an erroneous action) because of the latter’s residual activation. This effect of old (distractor) items on the selection process of the target goal is called the interference level. To be able carrying on with a task, it is generally preferable for the activation of old goals to decrease. However, the reactivation of a previous goal is sometimes necessary, for instance, when one needs to resume a task that has been interrupted. For the primary task goal to direct behavior again when the interruption ends, it must be reactivated above the interference level. This successful reactivation can occur if relevant cues are available to prime the target goal in the post-interruption environment. These cues can represent elements of the environmental context in which the ongoing task is taking place or elements of the mental context, such as knowledge about the task. Additionally, the retrieval of the primary task goal can be facilitated if its activation was boosted immediately before an interruption, for instance, when individuals are given the opportunity to prepare for the suspension of their task (Trafton et al., 2003).

Memory for goals has been extensively tested in various contexts and is able to account for numerous factors related to the impact of interruptions, the ways in which recovery can be facilitated, and the individual characteristics associated with effective task resumption (e.g., Trafton et al., 2003; Ratwani and Trafton, 2008; Morgan et al., 2013; Gartenberg et al., 2014; Foroughi et al., 2016c; Wilson et al., 2018). The great emphasis placed on interference by MFG goes hand in hand with the abovementioned findings showing that WMC is positively associated with interruption recovery. Individuals with greater WMC are presumably better able to prevent the goals from the interrupting task to interfere with those of the primary task during the interruption and upon resumption. However, some studies have revealed that the ability to maintain pre-interruption information in memory in spite of the potential interference caused by an interrupting activity could not fully explain how an interrupted task is resumed (Miller, 2002; Salvucci, 2010; Foroughi et al., 2016c; Labonté et al., 2016, 2019; Nicholas and Cohen, 2016). Some of these investigations have been conducted in static contexts, which suggests that even when pre- and post-interruption scenes are identical, cognitive processes other than working memory are involved in task resumption. Moreover, one can assume that other processes are even more critical in dynamic contexts, in which the continuous evolution of the state of the primary task makes the recall of the pre-interruption scene less central to the resumption process (Salvucci and Taatgen, 2011).

In comparison with static contexts, events in dynamic situations can occur in an independent manner, without the intervention of the individual performing the task. Therefore, the state of the primary task can evolve greatly during an interruption. Even if information pertaining to the pre-interruption scene can be easily retrieved from memory, it can turn out to be irrelevant once the interruption is over. In this regard, although it generally builds on the postulates of MFG, threaded cognition (Salvucci and Taatgen, 2011) recognizes the crucial importance of keeping primary task information active in memory for the resumption of static tasks only. In dynamic settings, this theory rather suggests that an individual must reconstruct the primary task context by visually examining the post-interruption environment to extract relevant information. Indeed, threaded cognition proposes that the information related to the pre-interruption state of the situation immediately becomes obsolete due to the evolution of the situation during the interruption. Yet, since few interruption studies have been performed using continuously evolving primary tasks, the precise nature of this reconstruction process remains unclear. Labonté et al. (2019) have shown that both the memory of the pre-interruption scene and the extraction of information from the post-interruption environment are central to recovery. However, when resuming a dynamic task, it can be argued that the importance of remembering pre-interruption information and preventing interference from interrupting events may be inversely proportional to interruption duration since the disparity between pre- and post-interruption scenes increases with the length of the interrupting task.

### The Role of Interruption Duration

The duration of an interrupting task is closely related to the severity of its impact on the resumption lag, which represents the time needed to perform the first action following an interruption (Hodgetts and Jones, 2006; Monk et al., 2008). The suggested explanation for this effect implies that the longer the interruption, the more the activation of information related to the primary task (i.e., the primary task goal in the MFG model) decreases in memory, whereas the more the activation of elements related to the interrupting task increases (Hodgetts and Jones, 2006; Foroughi et al., 2016c). Consequently, the reactivation of the information related to the primary task that is needed to resume this interrupted task is more time-consuming following long interruptions.

Foroughi et al. (2016c) have shown that a high WMC mitigated the negative impact of interruption length on task resumption. High-WMC individuals appear to be better able to prevent the decrease in activation of relevant primary task information and to deal with the growth of distracting information that are brought by prolonged interruptions. Their advantage in managing the consequences of lengthy interruptions was observed in static contexts, in which the primary task did not evolve no matter how long it was suspended. However, there is no certainty that a high WMC would still be helpful in dynamic contexts, especially in the case of long interruptions. Being able to maintain pre-interruption elements in memory despite the presence of irrelevant information may not prove as crucial in a situation that progresses even during an interruption, thereby potentially decreasing the distinction between low- and high-WMC individuals. Indeed, making use of information related to the pre-interruption state of a task is not necessarily the most efficient way to go on with a continuously evolving primary task following its suspension. It may rather be preferable to reconstruct the primary task context based on elements of the new task environment (see Salvucci and Taatgen, 2011).

Manipulating the duration of interruptions occurring in a continuously evolving context appears to be the ideal solution to study how dealing with the suspension of a dynamic task differs from managing interruptions in a static environment. Regardless of the nature of the task, pre- and post-interruption scenes will differ little following a short interruption. However, while lengthening the interruption will increase the discrepancy between pre- and post-interruption situations in dynamic settings, both scenes will always remain identical in static contexts. Therefore, varying interruption duration in an actively evolving environment can likely help qualify the interpretation of the differences between static and dynamic task resumption processes.

### The Current Study

The objective of the current study was to examine the role of working memory and reconstruction mechanisms in dynamic task resumption and to characterize the importance of their role following interrupting tasks of different lengths. We hypothesized that the relative contribution of working memory and reconstruction would vary according to the duration of the interruption, and therefore, the extent to which the primary task would evolve while it gave way to the interrupting activity. If working memory is more useful when pre- and post-interruption situations are similar, its contribution to interruption recovery should decrease as interruption length increases. In contrast, because extending the duration of an interruption increases the disparity between pre- and post-interruption scenes, the involvement of reconstruction in recovery should be greater following lengthy interruptions. To shed light on this issue, we employed an individual differences approach whereby we studied the association between (1) the ease with which participants recover from interruptions of various durations when carrying out a dynamic primary task and (2) performance on tasks aimed to reflect the two abovementioned processes of interest.

Our primary task consisted of a modified version of the multiple object tracking (MOT) paradigm (see Hunter and Parush, 2010). In this task, participants were presented with a set of dots that moved slowly but continuously on the screen. They were asked to visually follow target dots among distractor dots of a different color. At the end of a trial, all dots stopped moving and became indistinguishable. Participants then had to identify which dots were the targets. However, on some trials, an interrupting task was presented just before the response phase. All dots continued their trajectory during the presentation of the interrupting task, making it necessary for participants to try to extrapolate the new position of the targets. Participants were asked to respond as fast but also as accurately as possible after an interruption. Because the abilities associated with the time needed to resume a task differ from those explaining the accuracy with which this task is recovered (Bai et al., 2014; see also Tremblay et al., 2012), emphasizing the importance of both post-interruption accuracy and resumption lag allowed us to examine how each of these two established indices of interruption recovery was associated with the cognitive processes of interest at each interruption duration.

The contribution of working memory in recovery following the interruption of our dynamic task was assessed by measuring participants’ WMC. As mentioned previously, a widely accepted definition of WMC concerns the ability to use attention to maintain task-relevant goals active despite the presence of interfering information (Engle, 2002). The process of reconstructing the context of an interrupted task is deemed to depend on a visual scanning of the environment (see Salvucci and Taatgen, 2011). However, Salvucci (2010) states that “because of the close interdependence between task domain and reconstruction, there is no easy domain-general way of specifying reconstruction processes” (p. 92). Given the nature of our primary task, in which participants were required to discriminate between relevant and irrelevant dots following an interruption, the importance of reconstruction was examined by measuring participants’ visual search capacity. The latter refers to the ability to effectively allocate visual attention to relevant stimuli presented among distractors (Kane et al., 2006). As demonstrated by Kane et al. (2006), this ability is independent of WMC. We examined which of these cognitive skills were predominantly associated with which aspect of dynamic task resumption using four different interruption durations (none, 5, 15, and 30 s). To do so, two multilevel regressions were performed: one with accuracy and the other with resumption lag as the predicted variable. The predictors consisted of participants’ scores on each task measuring the two cognitive abilities of interest, the length of the interruption, as well as the interaction between each cognitive skill and interruption duration.

## Materials and Methods

### General Method

#### Participants

One hundred and seventeen students and employees at Université Laval received a CAD $10 compensation for their participation in a single 90-min experimental session. All participants reported normal or corrected-to-normal vision and hearing. The experiment was approved by the Ethics Committee of Université Laval and written consent was obtained from all participants prior to testing. Data from six participants (all women) regarding any of the three tasks were lost due to a technical problem with data saving. Consequently, all data from those participants were removed from the analyses. In addition, data from one male participant were removed from all analyses because of extreme response time values on the MOT task (see Data Processing and Analyses section below). The final set included 110 participants (42 men, mean age *=* 24.26 years), which is adequate to test a medium-sized relationship between five individual predictors and the dependent variable of a multiple regression assuming an alpha level of 0.05 and a statistical power of 0.80 (see Tabachnick and Fidell, 2013).

#### Materials

All tasks were controlled by a PC computer with a 23-inch monitor. The monitor had a resolution of 1,920 × 1,080 pixels and a refresh rate of 60 Hz. Participants were seated approximately 60 cm from the screen.

#### Procedure

As the main task in the current experiment, the MOT task was always performed first. The secondary tasks measuring WMC and visual search capacity were then performed, their order being counterbalanced across participants. Before each task, the experimenter gave a verbal explanation to the participants before letting them read written instructions at their own pace. The experimenter stayed with the participants for practice trials (except for the WMC task, which comprises numerous practice trials with very detailed instructions) and answered their questions before letting them execute the experimental trials alone in the soundproof room. Each experimental task is described in more detail below.

### Multiple Object Tracking

The primary task consisted of a modified version of the MOT task used by Hunter and Parush (2010). Participants had to visually pursue target dots among distractor dots. In some trials, the tracking task was interrupted by mathematical verifications. The dots continued their trajectory during the interrupting task. At the end of the interruption (or at the end of the movement phase in the absence of an interruption), participants had to indicate which dots they thought were the targets.

#### Materials and Stimuli

**Figure 1:**
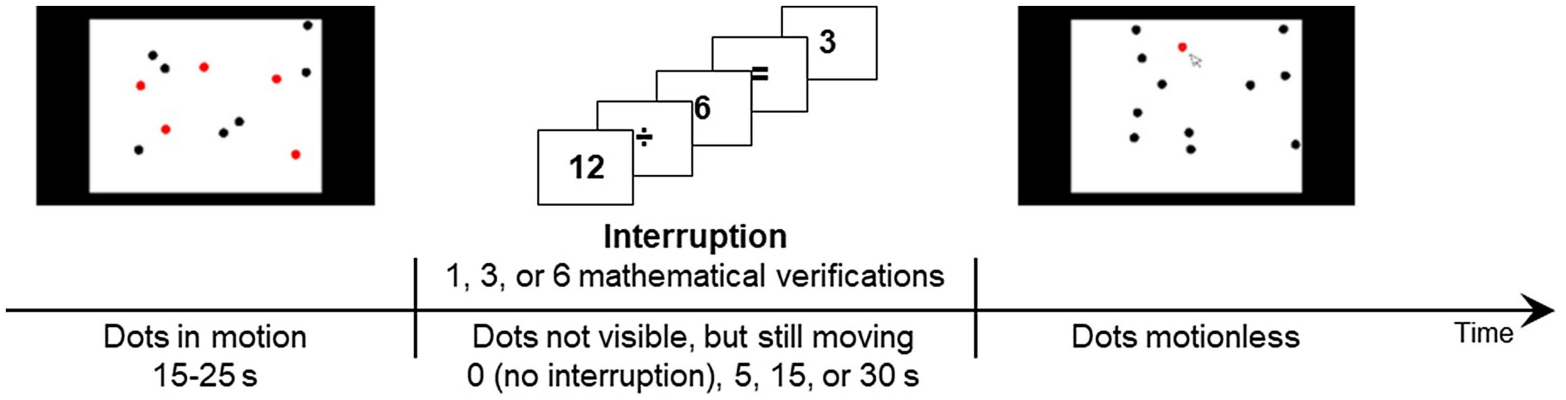
Schematic representation of the multiple object tracking task. The left part of the timeline represents the visible part of the movement phase. The middle part depicts the execution of the interrupting task (if any), during which the dots continued their trajectories despite not being visible. The right part of the timeline represents the response phase, in which all dots turned black and participants had to select those they thought were the targets.

The MOT task was controlled using Python 3.5. Twelve dots were presented simultaneously in a white rectangle display which measured 1,200 × 900 pixels (28.07° × 21.80°) and was centered on a black background. Out of the 12 dots, five consisted of red targets, whereas the remainder were black distractors (see Figure 1). Each dot had a diameter of 48 pixels (1.21°). The starting position of the dots was computed quasi-randomly, with the constraint that the minimal distance between the center of two dots or between the center of a dot and any side of the display was 60 pixels (1.53°). For target dots specifically, the minimal distance between their center was 120 pixels (3.05°). The direction in which each dot started moving was established randomly. During the movement phase, each dot moved at a constant speed selected randomly between 4.8 and 12 pixels/s (0.12°–0.30°/s, respectively). A dot bounced on one edge of the display when they both became separated by only one pixel. In that case, the dot changed its direction either in x or in y, according to whether it collided with a vertical or a horizontal wall, respectively. When two dots merging in the same direction became separated by only two pixels, their respective trajectory was swapped, which made them look like they bounced on each other.

For each problem of the interrupting mathematical verification task, participants were presented with two numbers separated by a mathematical operator (+, −, ×, or ÷) and with the symbol “=” followed by a suggested answer (either correct or incorrect; see Figure 1). For addition and subtraction problems, both terms ranged from 1 to 12. The first term of a subtraction was always greater than the second term to prevent the difference from being negative. For multiplication problems, both factors ranged from 2 to 10. For division problems, the dividend ranged from 8 to 49 and the divisor ranged from 2 to 10. The suggested results of the mathematical equations (whether correct or incorrect) were always integers.

#### Procedure

At the beginning of each MOT trial, a fixation cross appeared in the center of the screen for 250 ms. Immediately after its presentation, all dots (both red targets and black distractors) appeared on the screen and immediately started to move in a linear fashion. The dots moved visibly for a random duration of between 15 and 25 s. When they stopped moving, they all became black and participants had to select those they thought were the targets. The dots turned red once selected and participants were not given the possibility to unselect them. Two seconds after participants selected their fifth answer, all dots turned black before the correct target dots started blinking in green three times (500 ms on and 500 ms off). Participants then had to press the spacebar to begin the next trial. The response phase could be immediately preceded by a 5-s, 15-s, or 30-s interrupting mathematical task which hid the whole primary-task interface while the dots continued their trajectories. Nine MOT trials were created for the uninterrupted condition and each of the three interruption durations. Four of them (one for each interruption condition) were used as practice trials, whereas the remaining 32 (eight for each interruption condition) were experimental trials. All trials within the practice and experimental phases of the experiment were the same for all participants but were presented in a different random order.

In the interrupting task, participants completed simple mathematical verifications, each problem being presented for a fixed duration of 5 s. Therefore, one, three, or six problems were presented during 5-s, 15-s, and 30-s interruptions, respectively. Each mathematical problem started with a 50-ms blank after which the terms and symbols were presented sequentially for 500 ms each, for a total of 2,050 ms. An answer to the problem was then suggested, and participants had 2,950 ms to indicate whether it was correct or incorrect by pressing either the “C” or the “I” keys of the keyboard, respectively. When participants failed to answer before the time limit, the ongoing problem was considered as incorrect and the program went on to the next mathematical verification (except for the final problem where the primary task interface reappeared). Otherwise, participants were presented with feedback regarding the accuracy of their answers until the end of the 5-s period associated with each problem. It should be noted that participants were asked to keep their dominant hand on the computer mouse at all times and to answer the interrupting problems on the keyboard using the other hand. All participants were presented with the same 90 mathematical problems (10 in practice and 80 in experimental trials). As with the MOT trials, they were presented in a random order to each participant. Therefore, the mathematical problem(s) included in a given interrupted MOT trial could change across participants. The MOT task took about 35 min to complete.

#### Measures

The MOT task allowed for the computation of three dependent variables. The first variable, which we termed accuracy, refers to the mean number of correctly identified targets in each type of trial.^1^ The second variable consisted of the time (in ms) needed for the participants to select the first dot they considered as being a target. This duration was computed either from the moment the dots stopped moving (uninterrupted trials) or from the moment the primary task display reappeared (interrupted trials). For interrupted trials, this variable corresponds to the resumption lag (i.e., the time needed to resume the interrupted task). Even though the primary task was not suspended in uninterrupted trials, we decided to name this variable “resumption lag” in all conditions for the sake of brevity. Finally, although not a critical variable to answer our research questions, we computed performance on the mathematical verifications to determine whether participants performed the interrupting task properly. Mean performance in the final set of participants was 90.60% (SD *=* 6.00%, range *=* 67.50–100%), confirming that participants were actively engaged in the interruption during its presentation.

### Working Memory Capacity

Working memory capacity was assessed using the automated operation span (Aospan) task (Unsworth et al., 2005). Participants had to recall the serial order of sequences of letters while performing a distracting mental operation (i.e., a simple mathematical verification) as quickly as possible before each letter was presented.

#### Materials and Stimuli

The Aospan task was run using E-Prime 2.0 Professional with a resolution of 1,024 × 768 pixels. We used Foster et al.’s (2015) version of the task, which we translated into French. It should be noted that we increased the duration of the feedback displayed following each trial to make sure participants had enough time to read. The to-be-remembered letters were 1.15° in height. They were presented in the center of the screen and were randomly taken without replacement from the letter set F, H, J, K, L, N, P, Q, R, S, T, Y. The distractor task, which consisted of mathematical verification problems, involved multiplications, divisions, additions, or subtractions with digits from 1 to 9. Participants were presented with all the terms and symbols of the operation together on the screen [e.g., (4 × 2) + 6 = ?]. However, the suggested answer was presented on a subsequent screen along with clickable buttons labeled “true” and “false” (for a schematic representation of the task, see Figure 1A of Foster et al., 2015).

#### Procedure

To familiarize with both phases of the task separately, participants started by performing four letter-recall trials followed by 15 mathematical verifications. Next, they completed three practice trials which, as experimental trials, involved the presentation of a mathematical verification and a to-be-remembered letter in alternation. When presented with a mathematical operation, participants had to solve the problem before clicking anywhere on the screen using the mouse. They were then presented with a suggested answer and had to indicate whether it was true or false by clicking on the appropriate button. If participants failed to respond within the time limit (i.e., within 2.5 standard deviations of their average response time computed during the practice phase involving only mathematical verifications), the ongoing problem was considered as incorrect. When all the letters of a to-be-remembered sequence had been presented, the set of all possible letters was displayed in a 3 × 4 matrix. Using the mouse, participants had to select the letters that were part of the just-presented sequence in their order of presentation. Following the practice session, participants completed three experimental blocks, each comprising five randomly presented trials of 3, 4, 5, 6, or 7 letters in length. Therefore, 75 letters had to be recalled overall. Feedback regarding the number of letters correctly recalled and the number of mathematical errors in the just-completed trial was presented for 6 s at the end of the recall phase, along with the overall percentage of accuracy for the mathematical verification task. A blank screen was subsequently presented for 1 s, followed by the appearance of the first mathematical verification of the next trial. The 15 experimental trials were presented without interruption and participants were unaware that they were blocked. The Aospan task took about 20 min to complete.

#### Measures

The Aospan score was calculated by summing the number of letters in every perfectly recalled list. Therefore, a greater score reflected a greater WMC. Mean accuracy for the mathematical verifications in the final set of participants was 92.92% (SD *=* 5.56%, range *=* 62.67–98.67%), suggesting that there was no tradeoff between recall accuracy and performance on the distractor task.

### Visual Search Capacity

The visual search task was based on Kane et al.’s (2006; Exp. 2) spatial configuration search task. Participants were asked to determine whether a visual target stimulus was present among a set of similar but irrelevant visual stimuli.

#### Materials and Stimuli

**Figure 2:**
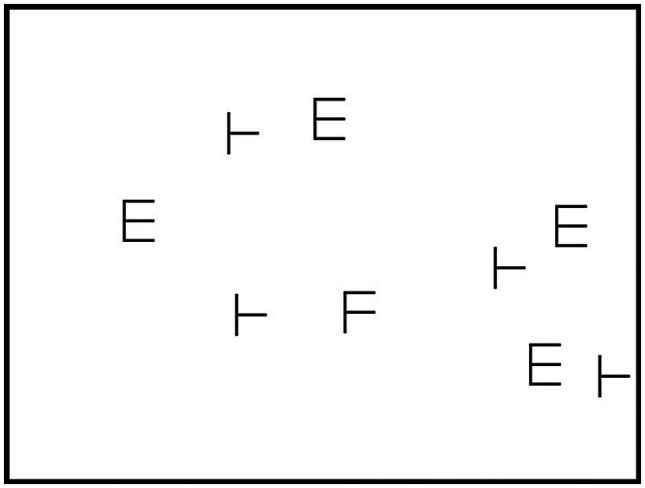
Screenshot of a medium-sized set of stimuli in the visual search task.

The visual search task was run using Python 3.5. The stimuli were presented in a white display with a dimension of 480 × 360 pixels (12.04° × 9.09°) that was centered on a black background. The target stimulus was the letter “F.” Distractor stimuli consisted of either the letter “E” or the letter “T” rotated 90° anticlockwise (see Figure 2). The target and distractor stimuli were created by drawing 32-pixels long vertical lines and 24-pixels long horizontal lines using Microsoft Paint. All lines were 2-pixels large. All stimuli measured 0.61° × 0.81°. The relative proportion of each type of distractor in each stimulus set (e.g., the number of “E”s compared to the number of “T”s) was determined randomly. The position of all items in the display was pseudo-random: Stimuli could not be vertically aligned and had to be separated by at least 11 pixels (0.28°) in all directions.

#### Procedure

At the beginning of each trial, a blank screen was presented for 490 ms, followed by a fixation cross displayed in the center of the screen for 740 ms. The stimulus array was presented immediately after the disappearance of the fixation cross. Participants were asked to indicate as fast but also as accurately as possible whether the target was present in the stimulus set. They were told to press the “Z” key of the keyboard if the target was present and to press the “M” key if the target was absent. No feedback was provided, and the next trial started immediately after participants gave their answers. Each trial was comprised of either 2, 3, 8, 9, 17, or 18 distractors. Furthermore, an additional stimulus (i.e., the target) could or could not be added to each of these sets of distractors, resulting in 12 different types of trials. Participants completed 24 practice and 96 experimental trials (comprising, respectively, two and eight trials of each type). The trials were the same across participants but were presented in a different random order. The visual search task took about 10 min to complete.

#### Measures

We computed mean accuracy and mean response time for all experimental trials, regardless of the set size and the presence or absence of a target. We used response time on correct trials (hereafter named correct RT) as our dependent variable of interest, a shorter correct RT denoting a greater visual search capacity. Mean accuracy for this task was 95.35% (SD *=* 4.31%, range *=* 72.92–100%).

### Data Processing and Analyses

#### Data Cleaning

To minimize the presence and impact of outliers in our time-based tasks, we identified the longest 1% response time data among all participants for each task separately. We then removed those trials from the analyses. More specifically, we removed the longest resumption lags overall for each condition of the MOT task and the longest response times overall for each set size of the visual search task. Despite the removal of the slowest response times observed in the sample, the mean resumption lag obtained by one participant for 5-s interruptions was still 3.29 SDs apart from the mean. This case was considered as an outlier (see Tabachnick and Fidell, 2013) and was therefore removed from all analyses. No other outlier cases were observed in comparison with the original means after removing the extreme response times. Furthermore, no outlier cases were present for accuracy in interrupted trials of the MOT task as well as for the Aospan score. The maximum percentage of trials that were withdrawn for a single participant in the final data set was 9.39% and 20.83% for the MOT and visual search tasks, respectively.^2^

#### Means Comparison

All analyses were performed using SPSS 27 (IBM) with an alpha level set to 0.05. Before examining the involvement of WMC and visual search capacity in dynamic task resumption, we first looked at global results on the primary MOT task to confirm the effect of the presence and the length of the interruption on both indicators of interruption recovery. For this purpose, a linear mixed model analysis with interruption duration (none, 5, 15, or 30 s) declared as a repeated effect was performed on both accuracy and resumption lag. The models were fit using restricted maximum-likelihood estimation, and the Satterthwaite approximation was used to calculate denominator degrees of freedom. The unstructured matrix was used as the covariance structure in both linear mixed models.

#### Multilevel Regressions

Next, we investigated the relationship between the two indices of recovery and each cognitive skill of interest. More importantly, we examined whether this relationship was moderated by the duration of the interruption. To that end, two multilevel regression analyses were used: one predicting accuracy and the other predicting resumption lag. Each model included WMC, visual search capacity, and interruption duration as predictors. In addition, the interaction between each cognitive ability and interruption duration was entered in the model to examine whether the role of WMC and visual search capacity varied at each interruption duration (none, 5, 15, or 30 s). All predictors were treated as continuous variables and were declared as fixed effects. The participant identification number was declared as a random effect to account for the non-independence of observations. As with the linear mixed models, the Satterthwaite approximation was used to calculate denominator degrees of freedom and the models were fit using restricted maximum-likelihood estimation.

## Results

### Global Results for the MOT Task

**Figure 3:**
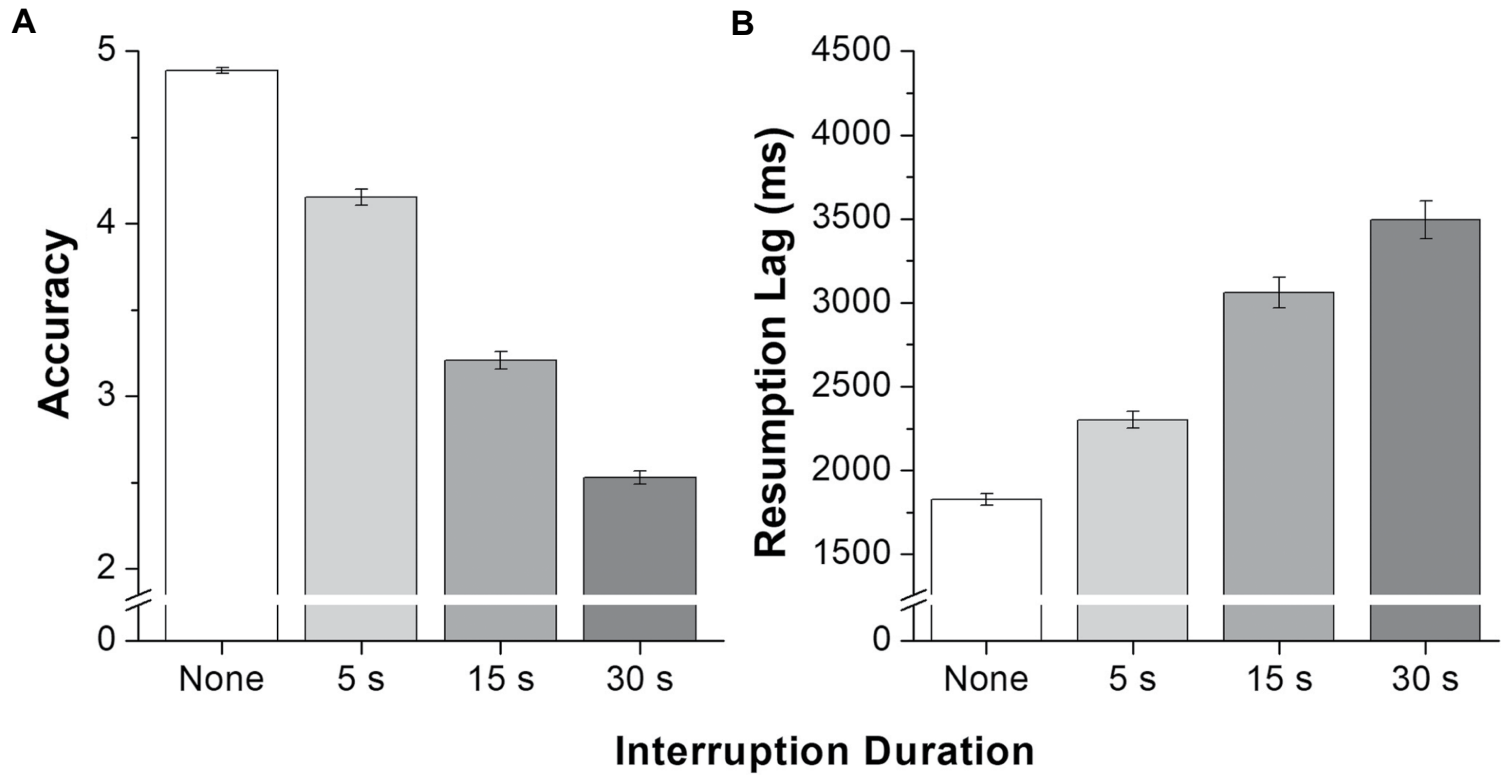
Mean number of target dots correctly identified **(A)** and mean resumption lag **(B)** according to interruption duration. Error bars represent the standard error of the mean.

As shown in Figure 3, the number of correctly identified targets in the primary MOT task was at its highest when no interruption occurred, and accuracy decreased as interruption length increased. Conversely, resumption lag increased with interruption duration, and resumption lag was fastest when no interruption occurred. The analyses confirmed this pattern of results by showing a main effect of interruption duration on accuracy, *F*(3, 109) *=* 1,325.28, *p* < 0.001, and resumption lag, *F*(3, 109) *=* 85.42, *p* < 0.001. Multiple comparisons revealed that accuracy and resumption lag differed between each interruption condition (all *p*s < 0.001). Such results confirm the expected effect of both the occurrence of the interruptions and their duration on interruption recovery. Despite the great difficulty of the task following a 30-s interruption, performance was still above chance level, which suggests that participants did not answer at random. Given the number of targets and distractors, random performance would have been 2.08 (Hulleman, 2005, p. 2300), whereas the mean performance in this condition was 2.53. A one-sample *t*-test showed that these two values were significantly different, *t*(109) *=* 12.07, *p* < 0.001.

### Global Results for Secondary Tasks

The mean Aospan score on the WMC task was 48.92 out of 75 (SD *=* 17.01), and the mean correct RT on the visual search task was 1,189.76 ms (SD *=* 220.55). A Pearson correlation indicated that the scores obtained by participants on these two tasks were not significantly associated, *r*(108) *=* −0.018, *p =* 0.851. Note that to facilitate the interpretation of the following regression analyses, we transformed participants’ correct RT into negative scores so that a greater score reflects a greater visual search capacity (e.g., 1,200 ms became −1,200 ms).

### Contribution of Each Cognitive Ability to Resumption

#### Accuracy

Table 1 presents the results of the multilevel regression performed on accuracy. Unsurprisingly, this analysis first showed that interruption duration was a predictor of the accuracy of post-interruption responses. As suggested by the negative coefficient, accuracy decreased as interruption length increased. WMC was also a significant predictor of accuracy. More precisely, accuracy increased with the score on the Aospan task (and therefore with WMC). This was true regardless of the length of the interruption, as the interaction between WMC and interruption duration was not significant. Visual search capacity did not predict post-interruption accuracy, whether alone or in interaction with interruption duration.

**Table 1:** Summary of the multilevel regression for accuracy.

Fixed effects	Estimate	SE	df	*t*	*p*
Intercept	4.434	0.219	259	20.21	<0.001
WMC	0.007	0.002	259	3.21	0.001
Visual search capacity	<|0.001|	<0.001	259	0.60	0.552
Interruption duration	−0.075	0.011	327	−6.73	<0.001
WMC × Interruption duration	<|0.001|	<0.001	327	−0.26	0.794
Visual search capacity × Interruption duration	<|0.001|	<0.001	327	0.02	0.987
Covariance parameters	Estimate	SE	*Wald Z*		*p*
Residual	0.181	0.014	12.79		<0.001
Intercept	0.036	0.012	3.08		0.002

#### Resumption Lag

**Figure 4:**
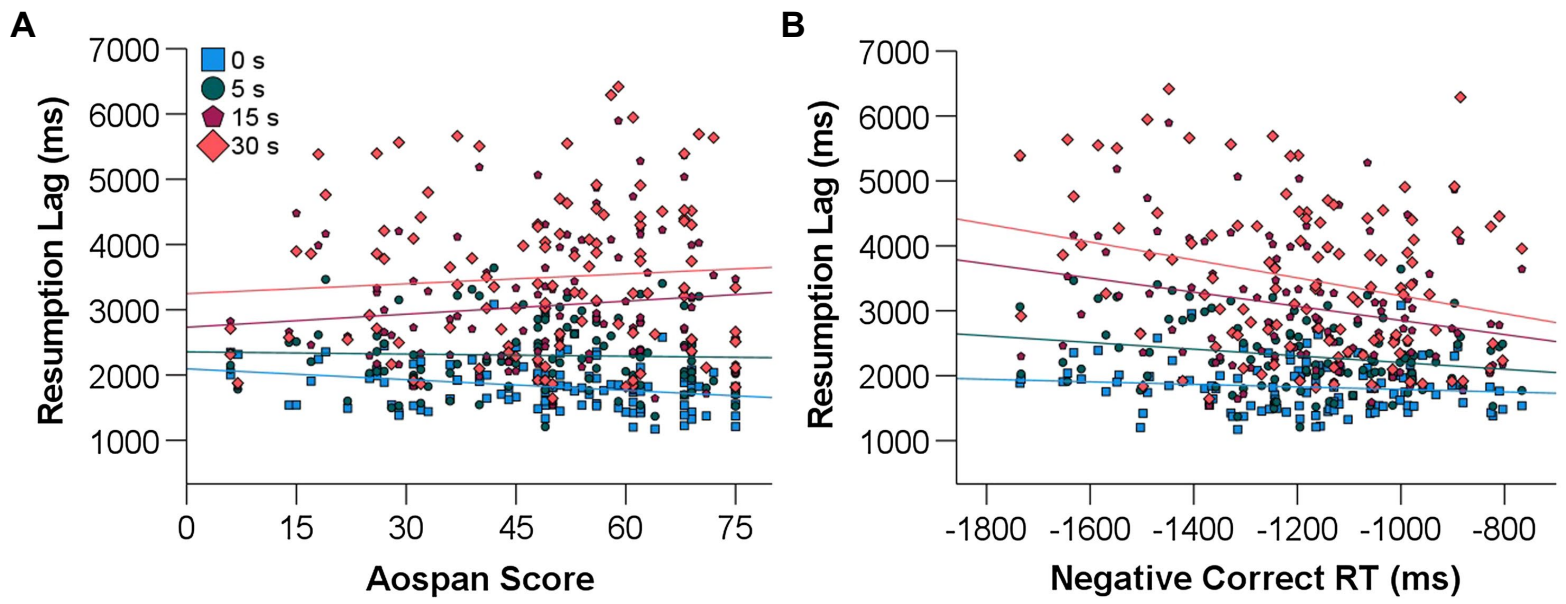
Relationship between working memory capacity (WMC; operationalized by Aospan Score) and resumption lag **(A)** and relationship between visual search capacity (operationalized by negative correct RT) and resumption lag **(B)** for each interruption duration (‘0 s’ corresponds to uninterrupted trials).

Table 2 presents the results of the multilevel regression conducted on resumption lag. Neither WMC, visual search capacity, or interruption duration alone was significantly associated with resumption lag. However, the interaction between WMC and interruption duration was significant, as was the interaction between visual search capacity and interruption duration. As indicated by the opposite direction of the estimated coefficients for the two interactions, interruption duration appeared to have a different impact on the relationship between both cognitive abilities and resumption lag. To better understand the observed interactions, four separate standard multiple linear regressions were performed to examine the relationship between, on the one hand, WMC and visual search capacity and, on the other hand, resumption lag in each interruption condition. In addition, Figure 4 illustrates the relationship between each cognitive ability and resumption lag according to interruption length.

**Table 2:** Summary of the multilevel regression for resumption lag.

Fixed effects	Estimate	SE	df	*t*	*p*
Intercept	1,775.391	413.994	184	4.29	<0.001
WMC	−2.993	3.901	184	−0.77	0.444
Visual search capacity	−0.301	0.301	184	−1.00	0.319
Interruption duration	−9.929	16.634	327	−0.60	0.551
WMC × Interruption duration	0.356	0.157	327	2.27	0.024
Visual search capacity × Interruption duration	−0.040	0.012	327	−3.28	0.001
Covariance parameters	Estimate	SE	*Wald Z*		*p*
Residual	406,642.816	31,801.985	12.79		<0.001
Intercept	257,123.229	49,692.074	5.17		<0.001

##### Uninterrupted Trials

The results of the multiple regression performed on baseline resumption lag (i.e., resumption lag on uninterrupted trials of the MOT task) are presented in Table 3. This analysis revealed that WMC was a significant predictor of resumption lag (*p* = 0.008). The observed negative relationship indicated that greater WMC was related to shorter resumption lag. Visual search capacity was not related to resumption lag in this context (*p =* 0.227).

**Table 3:** Summary of the standard multiple regression for resumption lag in uninterrupted trials.

Variable	*B*	SE B	*β*
WMC	−5.470^**^	2.008	−0.253
Visual search capacity	−0.188	0.155	−0.113
Adjusted *R*^2^	0.061		
Δ*R*^2^	0.078		
Δ*F*	4.51^*^		

* *p* < 0.05,

** *p* < 0.01

##### 5-s Interruptions

The results concerning the multiple regression performed on resumption lag following 5-s interruptions are presented in Table 4. The analysis showed that WMC did not predict resumption lag in this condition (*p* = 0.739). Although visual search capacity was associated with faster resumption (*p* = 0.026), the regression model was not significant (*p* = 0.077).

**Table 4:** Summary of the standard multiple regression for resumption lag following 5-s interruptions.

Variable	*B*	SE B	*β*
WMC	−0.984	2.943	−0.032
Visual search capacity	−0.513^*^	0.227	−0.213
Adjusted *R*^2^	0.029		
Δ*R*^2^	0.047		
Δ*F*	2.62		

* *p* < 0.05

##### 15-s Interruptions

With respect to 15-s interruptions (see Table 5), the analysis showed that greater visual search capacity was related to shorter resumption lag, and therefore faster resumption (*p =* 0.007). However, WMC did not predict resumption lag following 15-s interruptions (*p =* 0.184).

**Table 5:** Summary of the standard multiple regression for resumption lag following 15-s interruptions.

Variable	*B*	SE B	*β*
WMC	6.918	5.169	0.124
Visual search capacity	−1.098^**^	0.399	−0.255
Adjusted *R*^2^	0.062		
Δ*R*^2^	0.080		
Δ*F*	4.62^*^		

* *p* < 0.05,

** *p* < 0.01

##### 30-s Interruptions

Resumption lag in the 30-s interruption condition was also predicted by a single skill (see Table 6), with better visual search abilities being associated with faster recovery (*p =* 0.006). Consequently, WMC was not significantly related to resumption lag (*p =* 0.408).

**Table 6:** Summary of the standard multiple regression for resumption lag following 30-s interruptions.

Variable	*B*	SE B	*β*
WMC	5.360	6.447	0.077
Visual search capacity	−1.387^**^	0.497	−0.260
Adjusted *R*^2^	0.055		
Δ*R*^2^	0.073		
Δ*F*	4.20^*^		

* *p* < 0.05,

** *p* < 0.01

## Discussion

Given most of the studies about task interruption have been performed in a static context, the goal of the current investigation was to examine how working memory and reconstruction contributed to dynamic task resumption following interruptions of various lengths. These processes were operationalized using participants’ scores on tasks measuring WMC and visual search capacity, respectively. The main task consisted of a modified version of the typical MOT paradigm in which participants followed target dots that moved slowly but continuously, even when a 5-s, 15-s, or 30-s interrupting mathematical task was presented. The time needed to select the first dot (resumption lag) and the number of correctly identified targets following an interruption (or when the dots stopped moving in the absence of an interruption) were used as indicators of the ease with which the primary task was resumed. Unsurprisingly, our results showed that the longer the interruption, the greater the consequences on post-interruption accuracy and resumption lag. More importantly, we employed an individual differences approach to examine how WMC and visual search capacity were related to recovery as interruption duration increased.

The results revealed that WMC was positively associated with post-interruption accuracy in all interruption conditions, whether the trials were uninterrupted or interrupted for 5, 15, or 30 s. While WMC was not related to resumption lag in the presence of an interruption, it was negatively associated with resumption lag in uninterrupted trials. In other words, individuals with greater WMC selected the first target dot faster in the absence of an interruption. On the other hand, visual search capacity was not related to resumption lag in uninterrupted trials. In addition, the regression model regarding resumption lag in the 5-s interruption condition was not significant, although there was still a positive relationship between visual search capacity and the speed of recovery. Visual search capacity was the sole ability associated with faster resumption lag for 15-s and 30-s interruptions, its contribution to recovery being slightly greater for the longest interruption duration.

### Relationship Between Targeted Processes and Recovery

In this section, we offer potential explanations for the observed results based on the current state of knowledge about task interruptions and compare our findings with those from previous interruption studies. We also interpret our results in the light of the MOT literature to come up with a more comprehensive explanation for our findings.

#### Working Memory

We initially hypothesized that the contribution of WMC to recovery would decrease with interruption length since long task suspensions increase the disparity between pre- and post-interruption scenes. The results were not consistent with our hypothesis, at least not for the accuracy of responses. In accordance with studies conducted in static settings (e.g., Drews and Musters, 2015; Foroughi et al., 2016a), WMC was associated with better baseline accuracy on our dynamic primary task. In other words, when the dots moved visibly for the entire duration of the trial, target identification became more precise as WMC increased. More importantly, a positive association between WMC and accuracy was observed in all interruption conditions. Interruption duration did not interact with WMC, suggesting that the relationship between WMC and accuracy was similar for 0-s, 5-s, 15-s, and 30-s interruptions.

Given the continuous evolution of our dynamic primary task, many changes could occur in the monitored situation with the passage of time. Longer interruptions not only decreased the probability that participants would correctly remember the pre-interruption state of the situation, but also increased the divergence between the pre- and post-interruption locations of the targets and the possibility that the dots would change their trajectory by bouncing on each other or the sides of the display. It is therefore surprising that WMC contributed to post-interruption accuracy even after 30-s interruptions. Since the dots moved slowly, they were probably still rather close to their initial location at resumption, even in the event of a change in trajectory. Therefore, participants with greater WMC may have been advantaged by being better able to keep the representation of the pre-interruption scene active in memory despite the interference caused by the need to respond to the interrupting mathematical problems. Indeed, Keane and Pylyshyn’s (2006) results suggest that the ability to recover targets following brief interruptions strongly depends on the proximity of the items to their pre-interruption location (see also Franconeri et al., 2012). Overall, these results echoed Drews and Musters’ (2015) findings, which showed that WMC attenuated the consequences of suspending an ongoing static task on post-interruption accuracy.

As demonstrated by Foroughi et al. (2016c), the facilitating effect of WMC in a static context can also be reflected in the time required to resume an interrupted task. By manipulating the duration of the interruption of a procedural task, these authors observed that the increase in resumption lag brought by prolonged interruptions was smaller for high- compared to low-WMC individuals. In the current study, however, no relationship was found between WMC and resumption lag in the presence of an interruption. The fact that WMC was associated with accuracy but not with resumption lag in the interrupted conditions may indicate that accuracy is more related to the quality of the mental representation of the pre-interruption scene than to the effortful reconstruction process of the primary task context after the interruption. One unexpected result was that WMC was related to shorter resumption lags in the absence of an interruption. Since the task was very easy in the uninterrupted condition, fewer cognitive resources were presumably required than in the other types of trials, which may have led to more mind wandering in this condition (see Taatgen et al., 2021). Therefore, it could be hypothesized that participants with high WMC resumed the task faster when it required few mental resources because of their greater ability to block interference caused by their own thoughts. This capacity could have made them more focused when they needed to select the target dots, although this question would certainly deserve further investigation.

#### Reconstruction

The analyses showed that visual search capacity was beneficial to resumption following 15- and 30-s interruptions. These results were consistent with our hypothesis that the effective allocation of visual attention to relevant information would become more important for resumption with increasing interruption duration, although the contribution of visual search capacity to the regression model was only slightly superior in the 30-s than in the 15-s interruption condition. Nevertheless, these results further support the importance of visual scanning in the environment when pre- and post-interruption situations differ significantly (see Labonté et al., 2019). Despite the dynamic nature of the primary task, the mental representation of the pre-interruption situation was likely sufficient to recover from a 5-s interruption because the targets were still very close to their pre-interruption position. The benefits of visual search capacity for dynamic task resumption were, however, solely related to the speed of recovery. This may suggest that contrary to post-interruption accuracy, resumption lag is more related to the time-consuming process of reconstructing the primary task context than to the memory of the pre-interruption scene.

Salvucci (2010) states that “In reconstruction, the user visually re-encodes the task environment to reconstruct the task context immediately prior to interruption” (p. 89). In the current study, however, the post-interruption task context was not the same as the one preceding the interruption. It can thus be hypothesized that participants’ objective was not to reconstruct the task context as it was before the interruption, but rather as it had presumably evolved while they performed the interrupting task. The need to discriminate the five target dots from the seven distractors led us to suggest visual search capacity as an appropriate measure to reflect participants’ ability to perform this reconstruction. Indeed, this skill speaks to their capacity to select target information correctly and rapidly among distractors. Of course, participants likely needed to remember the pre-interruption location of the target dots first before they could choose target candidates. They may also have tried to use the dots’ pre-interruption trajectories to extrapolate their new position (Iordanescu et al., 2009; Hunter and Parush, 2010). The reconstruction of the task context may have been more efficient for participants with greater visual search capacity because they were faster in identifying potential target dots among distractors after attempting to extrapolate their new position. Whether individuals can predict the motion of tracked items during interrupted MOT trials is still a debated issue (see Meyerhoff et al., 2017). Some authors suggest that the ability to recover targets depends more on their proximity to their pre-interruption location than on the predictability of their trajectory (Keane and Pylyshyn, 2006; Franconeri et al., 2012). Nevertheless, other studies have shown that motion extrapolation was possible for a small number of items (Fencsik et al., 2007; Iordanescu et al., 2009; Hunter and Parush, 2010; Howe and Holcombe, 2012). Due to the nature of the task used here, it may be hypothesized that reconstruction could not have taken place without some representations of the pre-interruption state of the situation (i.e., pre-interruption locations and/or trajectories of the target dots). Indeed, in the post-interruption environment, all dots were identical and motionless. Therefore, in this specific context, it is possible that visual search capacity would not have been associated with faster resumption lag if the interruption was so long that participants were unable to retrieve anything from the pre-interruption scene.

Lastly, another factor could help explain the fact that visual search capacity was related to resumption lag for 15-s and 30-s interruptions, but not for 5-s interruptions. Indeed, the variability in resumption lag data was smaller in the latter condition than in the other interrupted trials. In fact, the variability in resumption lags following the 5-s interruptions was more similar to the variability in response times observed for uninterrupted trials than to that observed for the other interruption durations.^3^ This observation is consistent with the idea that there was little or no reconstruction when the target dots were invisible for only 5 s, as was presumably the case when the task was uninterrupted.

### Theoretical Implications

By helping to understand the characteristics associated with task resumption in dynamic settings, the results of the current study can help supplement the theories of task interruption, which have mainly been created to account for recovery in static settings. In the following section, we explain how our results relate to the two major theories of task interruption, namely, MFG (Altmann and Trafton, 2002) and threaded cognition (Salvucci and Taatgen, 2011).

#### Memory for Goals

The MFG model accounts for the delays and errors that can occur following the temporary suspension of the focal task by the fact that the goal needed to perform this task decays in memory during an interruption. This decay is deemed to take place to the benefit of the activation of interfering goals related to the interrupting activity. Our results regarding accuracy are consistent with the MFG model. WMC presumably helped participants recover from the interruptions by helping to strengthen the activation of information relevant to the primary task and to deal with the interference caused by the interrupting task. However, WMC was unrelated to resumption lag in interrupted trials. This particular result is not consistent with MFG: According to the model, WMC should not only be positively related to the relevance of the goal retrieved in memory (i.e., to post-interruption accuracy), but also to the time needed for this goal to be retrieved (i.e., to resumption lag).

Perhaps more importantly, the notion of goal activation that is at the core of the MFG model does not seem to apply to the paradigm used in the current study. Indeed, the main goal of the participants in our MOT primary task was to follow all target dots. It is hardly conceivable that they could have forgotten this goal after performing an interrupting task lasting up to 30 s, especially since all the dots reappeared on the screen after the interruption. In contrast with the tasks typically used in the interruption literature (e.g., the Tower of London task; Hodgetts and Jones, 2006), our dynamic primary task is hardly dividable into goals or sub-goals. Despite its divergence with other tasks used in interruption research, our MOT task may nevertheless be relevant for real-world activities that primarily require visual attention, such as security surveillance (e.g., Gill et al., 2005; Gill and Spriggs, 2005; Hodgetts et al., 2017). Theories should therefore be able to account for different types of tasks requiring different types of cognitive operations when explaining interruption recovery. In this regard, the threaded cognition theory seems to be able to explain a greater part of our results.

#### Threaded Cognition

Like MFG, threaded cognition generally gives an important role to the maintenance of primary-task information in memory for the resumption of an interrupted task. In fact, both theories share many similarities as their predictions are based on the Adaptive Control of Thought-Rational cognitive architecture (ACT-R; Anderson and Lebiere, 1998). However, rather than suspending the *goals* related to the primary task, threaded cognition posits that an interruption suspends the use of a resource called “problem state.” This resource shares its name with the information it is deemed to process, that is, the temporary information that must be kept in memory to be able to perform a task. Since the problem state resource cannot be used simultaneously by two activities, an interrupting task prevents the cognitive system from maintaining the problem state related to the primary task. Therefore, the activation of the primary-task problem state must be reinforced before, during, or immediately after an interruption through rehearsal or cueing to facilitate interruption recovery (similar to MFG’s propositions regarding the ways to keep target goals active in memory).

Conceptually, this notion of restoring a problem state rather than a goal is applicable to our MOT paradigm. Here, the problem state for the primary task may consist of the position and trajectory of the target dots. However, threaded cognition suggests that the primary-task problem state may quickly become irrelevant during the interruption of a continuously evolving task. In such a case, an individual must reconstruct the problem state by visually examining the post-interruption environment. As mentioned previously, in our MOT task, reconstructing the problem state could mean extrapolating the post-interruption position of the target dots based on their pre-interruption position and being able to differentiate targets from potentially near distractors. In this regard, the ability to effectively allocate visual attention to target information among distractors—namely, visual search capacity—appears to be essential for dynamic task resumption, at least when the pre- and post-interruption scenes differ greatly. However, to be able to account for the relationship we observed between WMC and accuracy, threaded cognition should further emphasize the importance of the representation of the pre-interruption scene in memory even in the presence of long interruptions occurring in a dynamic context (see also Labonté et al., 2019). Furthermore, while Salvucci and Taatgen (2011) suggested that the reconstruction process differs according to the task domain, our observation of a relationship between a general measure of, on the one hand, allocation of visual attention and, on the other hand, the ease with which a dynamic target tracking task was resumed evokes the possibility that visual search capacity may be useful for the reconstruction of any interrupted dynamic task that is visual in nature.

In summary, the current study shows that being able to keep the representation of the primary task active in memory seems to be important for dynamic task resumption even when the state of the primary task evolves during an interruption (see also Labonté et al., 2019). The current study also highlights the importance of visual search capacity for effective recovery following the suspension of a continuously evolving task, especially when encountering long interruptions. Given its emphasis on both memory and reconstruction processes, threaded cognition appears to be the most appropriate theory to account for dynamic task resumption. However, while threaded cognition suggests that information regarding the pre-interruption scene may quickly become obsolete after the interruption of a dynamic task, our results show that working memory can still significantly contribute to the resumption of an actively evolving activity.

### Future Research

Even in situations that do not evolve, working memory alone cannot explain interruption recovery (Foroughi et al., 2016c). A deeper examination of the importance of visual search capacity is therefore an interesting avenue to target the processes at play when resuming interrupted static tasks. Indeed, threaded cognition suggests that reconstruction occurs whenever the information pertaining to the pre-interruption scene cannot be retrieved, whether because this information was too complex or because the interruption was too long. Making a direct comparison between steady and continuously evolving situations would be an interesting way to better understand how different static and dynamic task resumption are. Such a comparison could help distinguish the importance for the recovery process of extrapolating the location of the target stimuli from that of remembering the pre-interruption state of the situation. To this end, it could be possible, for instance, to make the evolution of the primary task either cease or continue during interruptions of various lengths occurring within a single experiment.

Threaded cognition’s problem state resource can only be used by a single task at once. Because the interrupting mathematical task of the current study necessitated the processing of problem-state information, it would be interesting to determine whether WMC would be even more strongly related to recovery with a blank interruption, that is, an interruption in which no specific cognitive operation must be performed while the primary task is suspended (e.g., because the interrupting event consists of the presentation of a blank screen; see Morgan and Patrick, 2013; Borst et al., 2015; Wilson et al., 2018). In addition, with an MOT primary task, the use of blank interruptions could facilitate recovery by maximizing the probability of occurrence of an “online” extrapolation of the position of the target dots, which seems possible for a small number of occluded targets (see Fencsik et al., 2007; Hunter and Parush, 2010).

## Conclusion

The current study aimed to better define the role of working memory and reconstruction in the effective resumption of an interrupted dynamic task. These concepts were operationalized using measures of WMC and visual search capacity. Our results suggest that, as in static contexts, WMC plays a great role in interruption recovery in dynamic settings. Its role appears to be similar regardless of interruption duration. On the other hand, the importance of visual search for recovery seems to be greater following long interruptions. To our knowledge, this study is the first to demonstrate that the contribution of cognitive processes to dynamic task resumption can vary according to interruption duration. These results help supplement current theoretical models of task interruption by highlighting the importance of considering not only the length of the interruption, but also the context in which it takes place before drawing conclusions about the mechanisms involved in resumption. Moreover, this study suggests that being able to quickly extract visual information from the post-interruption scene is a more important predictor of recovery than WMC when considering the typical indicator of recovery (i.e., resumption lag) in a dynamic context. Such results highlight the need to update theoretical models of task interruption to better account for dynamic task resumption, presumably by giving a greater role to processes other than the sole retrieval of the pre-interruption state of the situation in the explanation of interruption recovery.

## Data Availability Statement

The datasets presented in this study, the SPSS syntaxes, and the code for the experimental tasks can be found at Open Science Framework: doi: 10.17605/OSF.IO/R2XHC.

## Ethics Statement

This study involving human participants was reviewed and approved by the Comité d’éthique de la recherche avec des êtres humains de l’Université Laval (2014-133 A-1/13-05-2015). The participants provided their written informed consent to participate in this study.

## Author Contributions

KL designed the experiment, supervised data collection, analyzed and interpreted the data, and wrote the manuscript with input from FV. FV helped elaborating the research questions and experimental design and revised the manuscript. All authors contributed to the article and approved the submitted version.

### Conflict of Interest

The authors declare that the research was conducted in the absence of any commercial or financial relationships that could be construed as a potential conflict of interest.

## Footnotes

^1^On two occasions (i.e., for one trial of the 5-s interruption condition of a participant and one trial of the 30-s interruption condition of another participant), accuracy was computed out of four instead of five dots. This was done because those two participants reported being unable to select the fifth dot of their choice, which seemed to be due to an infrequent problem with the task.

^2^In the visual search task, 20.83% of the trials were removed for two participants. For the next participant with the most trials withdrawn, 10.42% of the trials were removed.

^3^The coefficient of variation for uninterrupted trials and trials interrupted during 5, 15, and 30 s was 20.14, 23.05, 30.99, and 33.71%, respectively.
